# GlnR Negatively Regulates the Transcription of the Alanine Dehydrogenase Encoding Gene *ald* in *Amycolatopsis mediterranei* U32 under Nitrogen Limited Conditions *via* Specific Binding to Its Major Transcription Initiation Site

**DOI:** 10.1371/journal.pone.0104811

**Published:** 2014-08-21

**Authors:** Ying Wang, Chen Li, Na Duan, Bin Li, Xiao-Ming Ding, Yu-Feng Yao, Jun Hu, Guo-Ping Zhao, Jin Wang

**Affiliations:** 1 Laboratory of Synthetic Biology, Institute of Plant Physiology and Ecology, Shanghai Institutes for Biological Sciences, Chinese Academy of Sciences, Shanghai, China; 2 Department of Microbiology, School of Life Science, Fudan University, Shanghai, China; 3 Laboratory of Physical Biology, Shanghai Institute of Applied Physics, Chinese Academy of Sciences, Shanghai, China; 4 Department of Medical Microbiology and Parasitology, School of Medicine, Shanghai Jiao Tong University, Shanghai, China; 5 Department of Microbiology and Li Ka Shing Institute of Health Sciences, The Chinese University of Hong Kong, Prince of Wales Hospital, Shatin, New Territories, Hong Kong SAR, China; University Paris South, France

## Abstract

Ammonium assimilation is catalyzed by two enzymatic pathways, i.e., glutamine synthetase/glutamate synthase (GS/GOGAT) and alanine dehydrogenase (AlaDH) in *Amycolatopsis mediterranei* U32. Under nitrogen-rich conditions, the AlaDH pathway is the major route for ammonium assimilation, while the GS/GOGAT pathway takes over when the extracellular nitrogen supply is limited. The global nitrogen regulator GlnR was previously characterized to activate the transcription of the GS encoding gene *glnA* in response to nitrogen limitation and is demonstrated in this study as a repressor for the transcription of the AlaDH encoding gene *ald*, whose regulation is consistent with the switch of the ammonium assimilation pathways from AlaDH to GS/GOGAT responding to nitrogen limitation. Three transcription initiation sites (TISs) of *ald* were determined with primer extension assay, among which transcription from *ald*P2 contributed the major transcripts under nitrogen-rich conditions but was repressed to an undetectable level in response to nitrogen limitation. Through DNase I footprinting assay, two separate regions were found to be protected by GlnR within *ald* promoter, within which three GlnR binding sites (*a1*, *b1* sites in region I and *a2* site in region II) were defined. Interestingly, the major TIS *ald*P2 is located in the middle of *a2* site within region II. Therefore, one may easily conclude that GlnR represses the transcription of *ald via* specific binding to the GlnR binding sites, which obviously blocks the transcription initiation from *ald*P2 and therefore reduces *ald* transcripts.

## Introduction

Nitrogen is present in nearly all bacterial metabolites and can be assimilated from inorganic or organic sources. To cope with variations of nitrogen sources (excess *versus* limitation), bacteria have developed sophisticated regulation systems for nitrogen assimilation. For most bacteria, like *Escherichia coli*, *Corynebacterium glutamicum* and *Streptomyces coelicolor*, the glutamate dehydrogenase (GDH) pathway is the major route for ammonium assimilation when bacteria are growing under ammonium-excess conditions, and the glutamine synthetase/glutamate synthase (GS/GOGAT) pathway then takes over when the environmental nitrogen supply is poor [1]. However, in *Amycolatopsis mediterranei*, an important actinomycete for industrial production of rifamycin, although the assimilation pathway is the same as most bacteria under nitrogen limitation, *i.e.* using the GS/GOGAT pathway, the alanine dehydrogenase (AlaDH) pathway, instead of GDH pathway, is responsible for ammonium assimilation under high ammonium conditions [2]. AlaDH catalyzes the reaction between pyruvate and ammonium to generate alanine without any expense of ATP. Similar to GDH, AlaDH also has a high K_m_ value for ammonium, and thus the AlaDH pathway is less efficient than the energy-consuming GS/GOGAT pathway in ammonium assimilation and is only suitable for bacteria growing in ammonium-excess conditions [2].

In enteric bacteria, two major regulators, NtrC and Nac, regulate the nitrogen assimilation in response to changes of ammonia concentrations [3]–[5]. Unlikely, in Gram-positive bacteria, global regulators are often employed to govern the genes involved in global nitrogen metabolisms, *e.g.* GlnR and AmtR. Take GlnR for example, in the model actinomycete *S. coelicolor*, Tiffert *et al.* [6] found that GlnR is a global regulator for nitrogen assimilation, which recognizes a 22-bp consensus sequence of gTnAc-n_6_-GaAAc-n_6_ that is comprised of an “*a*-site” of “gTnAc” and a “*b*-site” of “GaAAc”. When the nitrogen supply is limited, GlnR acts as both an activator and a repressor, *e.g.* GlnR switches on the GS/GOGAT pathway *via* activating the transcription of *glnA* and switchs off the GDH pathway at the same time through repressing the *gdhA* transcription. Similarly, in *Streptomyces venezuelae* and *Mycobacterium smegmatis*, GlnR is also found to be a global regulator for the nitrogen metabolisms [7]–[9]. In *Amycolatopsis mediterranei*, enzymes involved in nitrogen metabolism with poor nitrogen sources, *e.g.* nitrate/nitrite redutases [10] and glutamine synthetase [11] are all stringently regulated by GlnR; however, little is known about AlaDH, which is the only known enzyme responsible for ammonium assimilation under nitrogen-rich conditions. Here in this study, we prove that the transcription of the AlaDH-encoding gene *ald* is directly negatively regulated by GlnR in *A. mediterranei* under nitrogen-limited conditions. Moreover, the in-depth mechanism for this transcriptional repression is also proposed.

## Materials and Methods

### Bacterial strains and media and primers

*A. mediterranei* strains, including the wild type, the *glnR* null mutant strain Rk [11] and the *glnR* complementation strain LRS987 (named as Rk/pVKER in [11]), were grown at 30°C in either Bennet rich medium [12] or minimal medium [13] with appropriate nitrogen sources supplemented [10]. When needed, erythromycin (200 µg/ml) and ampicillin (100 µg/ml) were added to the media. Primers were listed in Table 1.

**Table 1:** Primers used in this study.

Primer Name	DNA sequences (5′-3′)
**For EMSA**	
aldbs_F2	TCGACGTGACCACCCGCT
aldbs_A	GCCTCCTGGGTCGGTCTG
**For DNase I footprinting assay and primer extension assay**	
U32aldp_F1	CGGGCGGTACCTCGAACA
ald_pe	GGAACGGCGATACGCACGGT
**For Northern blot**	
aldN	ACCGTGCGTATCGCCGTTC
aldC	AGGCGAGGACGGTGTCCAG
**For RT-PCR**	
rpoB_f	CGTCTACTACTCCAAGGACA
rpoB_r	GTAGTCGATCTGGTCGGTGA
ald_f	GAGCTCATCCTCAAGGTCAA
ald_r	AGTAGAAGACGGACTCGTGC
glnR_f	ACCGTGACGTGATCCTCCTC
glnR_r	CTTCGTCGATCACCAGCTCA

### Total RNA isolation, Reverse Transcription-PCR (RT-PCR) and Northern Blot

*A. mediterranei* strains were all grown on Bennet agar supplemented with either 120 mM ammonium or 80 mM nitrate for 4 to 5 days. Total RNA was extracted from the mycelia using TRIzol reagent, following the manufacture's instructions, while RT-PCR assay was performed as described before [14]. Northern analyses were carried out as described [15], using a 1% agarose formaldehyde gel. Total RNA was extracted from U32 that was grown in minimal medium with different extracellular nitrogen sources supplemented. Fifty microgram total RNA was loaded onto each gel slot after heating at 65°C for 10 min and quickly chilling on ice to destroy the secondary structure. A DNA fragment containing the whole *ald* gene (1.1 kb) was used as a template to prepare probes by random-primed α-^32^P-dCTP labeling. Hybridization and washing procedures were performed at 42°C at the presence of 50% de-ionized formamide.

### Primer extension analysis

*A. mediterranei* U32 was grown in Bennet liquid medium supplemented with different extra nitrogen sources and total RNA was extracted for primer extension assay. According to the method previously mentioned [16], 20 µg total RNA was used in each assay, using the γ-^32^P-labeled primer ald_pe, which was complementary to the −3th to the 17th nucleotides of the *ald* protein coding sequence (CDS). At the same time, radio-labeled ald_pe was also used for preparation of the sequencing ladder, with the usage of the fmol DNA Cycle Sequencing System (Promega). The reverse transcribed products together with the sequencing ladders were analyzed on 6% ployacrylamide sequencing gels containing 7 M urea and analyzed with a phoshorimager.

### Electrophoretic Mobility Shift Assay (EMSA) and DNase I footprinting assay

The *ald* promoter region was PCR amplified, purified with gel purification kit and then labeled with γ-^32^P-ATP using T4 polynucleotide kinase (PNK) (NEB). Production of purified recombinant *A. mediterranei* GlnR as well as the EMSA conditions were the same as reported [10]. For DNase I footprinting experiment, primer ald_pe was firstly end-labeled with γ-^32^P-ATP using T4 PNK, and then a 527-bp DNA fragment was PCR amplified using primer U32aldp_F1 and γ-^32^P-labeled primer ald_pe. Proper probe (about 120,000 cpm) was incubated at 30°C for 30 min with different amounts of purified His_6_-GlnR, 1 µg sheared salmon sperm DNA in a total volume of 40 µl in the same buffer as EMSA and 0.3 Unit DNase I (TaKaRa, Shiga, Japan) was used for digestion at room temperature for 1 min. The preparation of DNA sequencing ladders and analyses of the digests were the same as reported before [16].

### Atomic force microscopy (AFM) imaging

The *A. mediterranei* U32 chromosomal DNA was used as a template for PCR amplification with paired primers aldbs_F2 and ald_r and the products (fragment AFM_Ald) were then purified for further AFM experiments. In a volume of 15 µl, 600 ng of fragment AFM_Ald was incubated for 15 min at room temperature with 1 µg of purified His_6__GlnR in the same binding buffer as used in EMSA. The reaction mixture was firstly diluted by 15 to 20 times with deionized water, and was then deposited on a piece of mica modified by Ni^2+^ and incubated for about 2 min. After gently washed with deionized water, the resulting sample was imaged with AFM. All images were performed in tapping-mode™ using a Multi-mode AFM with a Nanoscope IIIa controller (Digital Instruments (D.I.) Co., Santa Barbara, CA, USA). AFM images were collected using commercialized silicon tip (NASC18, MicroMasch) with a spring constant of 3.5 N/m and a resonant frequency of 75 kHz. All AFM experiments were conducted in an ambient environment.

## Results

### GlnR negatively regulates the transcription of *ald* in *A. mediterranei* U32

**Figure 1:**
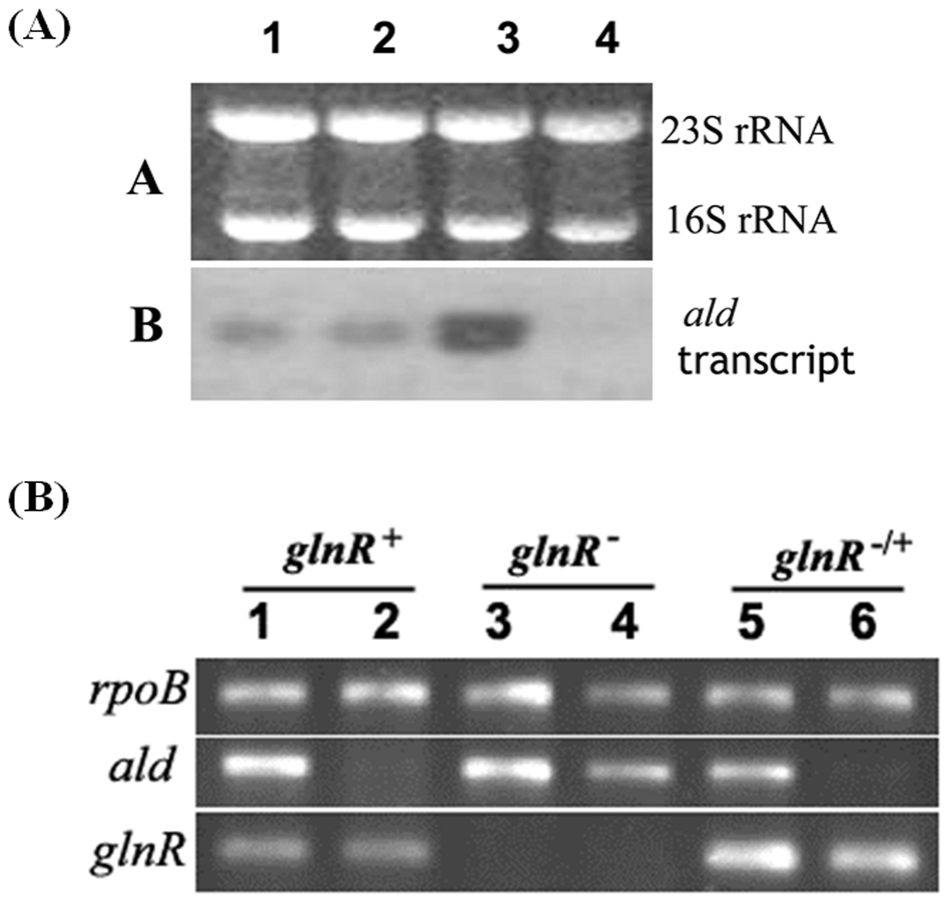
Analysis of the *ald* transcripts in *A. mediterranei*. (A) The *A. mediterranei* U32 *ald* transcripts analysed by Nothern Blot. U32 was cultured in MM supplemented with either ammonium sulphate or potassium nitrate. Lane 1, MM with 30 mM ammonium; Lane2, MM with 60 mM ammonium; Lane 3, MM with 120 mM ammonium; Lane 4, MM with 80 mM nitrate. Approximately 5 µg RNA was loaded onto each lane and the amount of RNA was visualized by ethidium bromide staining. In experimental group, 50 µg RNA of each sample was used for Northern hybridization. (B) RT-PCR analysis for *ald* and *glnR* mRNA levels in different *A. mediterranei* strains cultured under either nitrogen-excess or nitrogen-limited conditions. Symbols used for RT-PCR analysis: *glnR* ^+^, wild type U32; *glnR* ^−^, Rk; *glnR* ^−/+^, LRS987. Lanes 1, 3 and 5, Bennet medium with 120 mM ammonium; Lanes 2, 4 and 6, Bennet medium with 80 mM nitrate.

To examine the expression of *ald* in response to extracellular nitrogen supplies, U32 was cultured in minimal medium supplemented with different nitrogen sources, including increasing concentrations of ammonium and nitrate, and total RNA was then extracted for Northern blot analysis (Fig. 1A). According to the Northern blot results, the transcription of *ald* increased with the increase of extracellular ammonium concentrations while was stringently repressed by nitrate, which usually represents a poor nitrogen source, demonstrating that *ald* transcription was either activated in nitrogen-rich conditions or repressed in nitrogen-limited conditions.

Similar results were obtained with RT-PCR assays when U32 was grown in rich Bennet medium supplemented with either 120 mM ammonium or 80 mM nitrate, *i.e.* transcriptional level of *ald* was high in ammonium conditions while no transcripts could be detected in the presence of nitrate. When reverse transcriptase was omitted from the RT reaction mixture, no visible DNA bands of PCR products were detected, indicating no contamination of DNA in the RNA samples (data not shown).

Besides *ald*, the transcriptional regulation of other nitrogen metabolism-related genes, including *glnA*, *nas* operon [17] and *amtB* (data not shown), in Bennet medium with ammonium (or nitrate) is the same as that in minimal medium with ammonium (or nitrate). Therefore, Bennet medium, which is easier to prepare, is usually used for analysis of gene transcription in U32 and Bennet medium supplemented with 120 mM ammonium is designated “nitrogen-rich” medium for U32 while supplemented with 80 mM nitrate is designated “nitrogen-limited” medium for U32.

In *glnR* null mutant Rk, the transcription of *ald* remained at a high level under nitrogen-limited conditions (Figure 1B, lanes 3 and 4). When *glnR* gene with its original promoter was introduced back into Rk, the transcriptional repression by nitrate supplementation reappeared again (Figure 1B, lanes 5 and 6), which thus indicated GlnR as a repressor for *ald* transcription in response to limitation of extracellular nitrogen sources.

### *ald* has three transcription initiation sites

**Figure 2:**
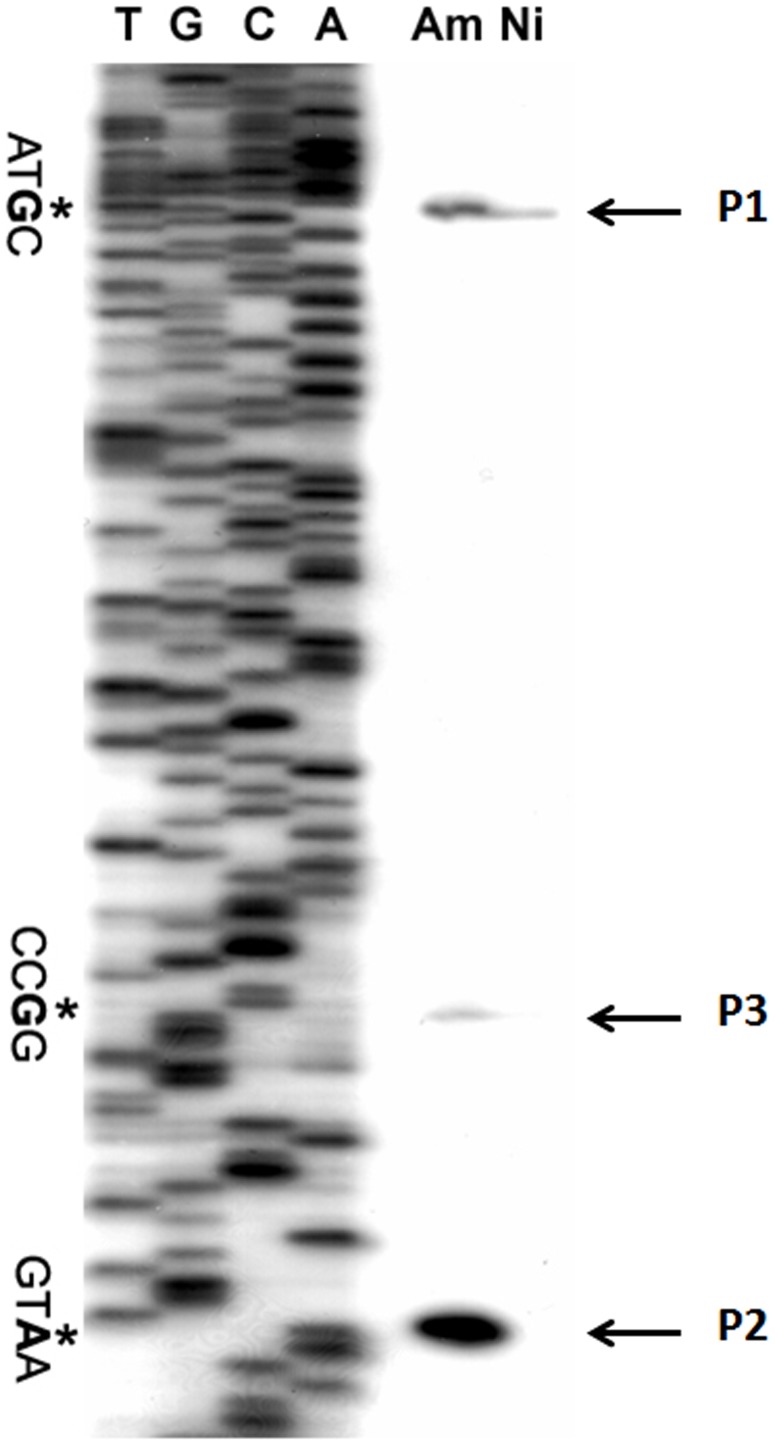
Identification of the transcription initiation sites (TISs) for *ald*. Total RNA was isolated from cultures in Bennet medium with different nitrogen sources, i.e., Am, 120 mM ammonium; Ni, 80 mM nitrate. TISs identified by the primer extension analysis were marked with asterisks and named as P1, P2 and P3, respectively.

Primer extension assay identified three transcription initiation sites (TISs) for *ald*, the −173G (P1), the −46A (P2) and −67G (P3) (relative to translation start site, Figure 2), among which P1 and P2 were main TISs while transcription from P3 was extremely weak and was only detectable under nitrogen-rich conditions (Figure 2). Under the nitrogen-rich condition, transcripts initiated from P2 contributed to the majority of *ald* mRNA, while the transcript from P2 was basically undetectable under nitrogen-limited condition (Figure 2), suggesting a stringent regulation of the transcription from P2 in response to extracellular nitrogen supplies. Compared to P2, the level of transcription from P1 was much weaker under the nitrogen-rich condition and obviously decreased, but to a detectable level under the nitrogen-limited condition. Therefore, P1 seems to be responsible for the basal level expression of *ald* that may be important for normal growth.

### GlnR protects two separate regions within the promoter region of *ald*

**Figure 3:**
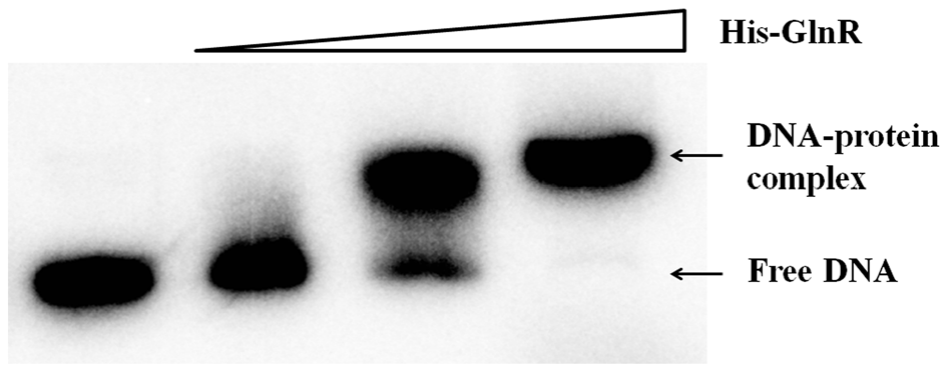
EMSA analysis of *ald* promoter with purified His-tagged GlnR. The promoter region of *ald* was amplified with primers aldbs_F2 and aldbs_A. Gamma-^32^P labeled DNA probe (0.04 pmol) was incubated with various amounts of purified GlnR (0 µg in lane 1, 0.4 µg in lane 2, 0.8 µg in lane 3 and 1.6 µg in lane 4). Two micrograms of sheared salmon sperm DNA was added to prevent nonspecific binding.

**Figure 4:**
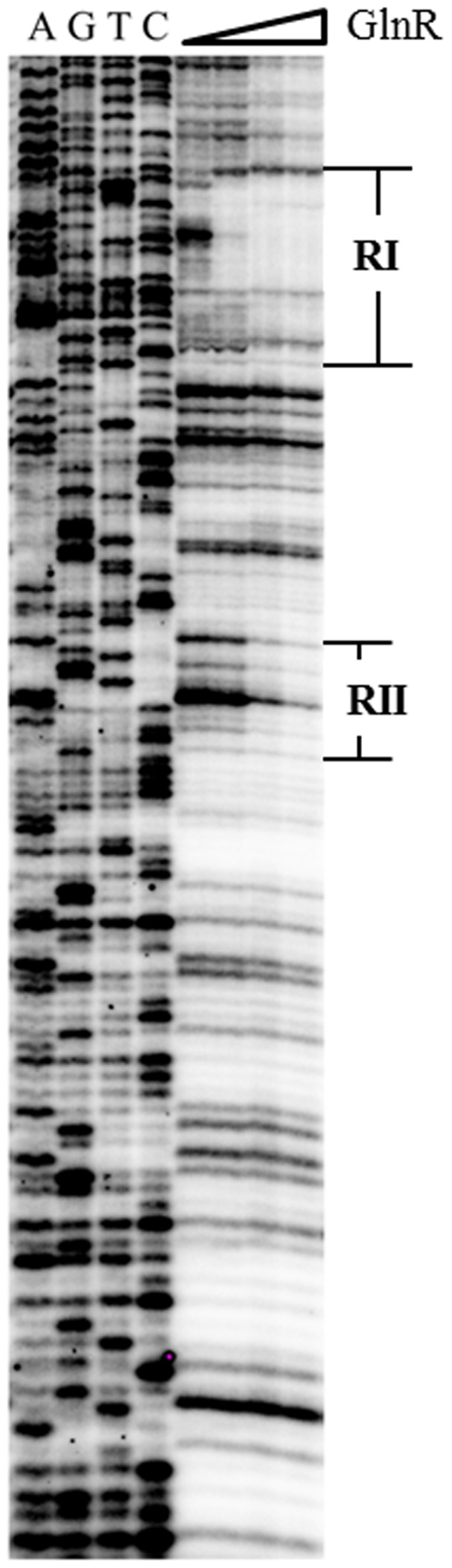
DNase I footprinting identification of the GlnR binding *cis*-element in *ald* promoter region. Different amounts of GlnR (0 µg in lane 1, 3 µg in lane 2, 6 µg in lane3 and 9 µg in lane4) were incubated with the labeled probes and DNA sequences protected by GlnR binding from DNase I cleavage were indicated (RI & RII).

With purified recombinant N-terminal His-Tagged GlnR and the *ald* promoter region, EMSA was employed to find that GlnR could specifically bind to the *ald* promoter *in vitro* (Figure 3). DNase I footprinting assay was further performed to characterize the precise DNA sequences that GlnR protected in *ald* promoter region, with two separate regions of RI and RII characterized (Figure 4). During the increase of GlnR concentrations, RI was apparently protected prior to RII, indicating GlnR has a higher binding affinity for RI (Figure 4).

**Figure 5:**
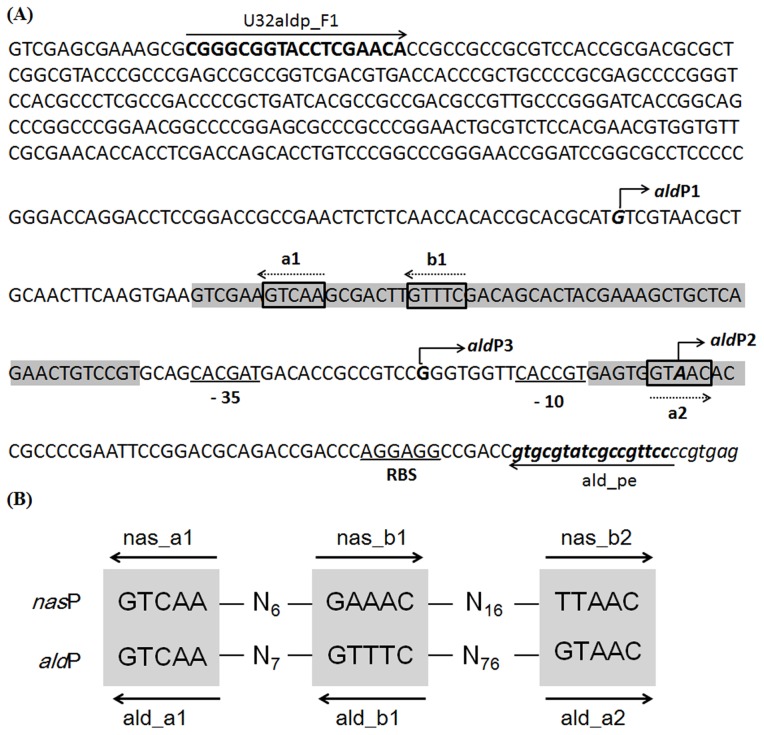
Manual definition of the GlnR binding sites in *ald* promoter (A) and comparison to those in *nasA* promoter (B). (A) The GlnR protected regions were shaded and putative *a*, *b* sites were boxed with their directions indicated by dashed arrows. Three transcription initiation sites *ald*P1, *ald*P2 and *ald*P3 were shown in bold and indicated by bent arrows. Putative −10 and −35 sequences, RBS (ribosome binding site) and the primer sequences used for DNase I footprinting assay were all labeled. (B) Comparison of the GlnR binding sites in p*ald* with those in p*nasA*. The arrows indicated the direction of the sites.

Three GlnR binding sites (*a1*, *b1* sites in RI and *a2* site in RII) were manually defined within the *ald* promoter region (Figure 5A), among which the DNA sequences of *a1* and inverted *b1* were identical to those of *a1* and *b1* sites in the promoter region of *nas* operon (Figure 5B) [10], and *a2* site of “GTAAC” followed the rule of “gTnAc” for an *a*-site as previously defined by Tiffert *et al.* [6]. The arrangement of the GlnR binding sites is similar in both promoters (Figure 5B), *i.e. a1*-*b1* sites are close in distance, while the third site (*ald_a2* or *nas_b2*) is separated from the *a1-b1* sites. In *nas* promoter, the internal distance between *b1* and *b2* sites is 16 bps [10], while *b1* and *a2* sites are separated by 76 bps in *ald* promoter.

Because the *ald* major transcription initiation site P2 locates in the middle of the *a2* site of “GT*A*AC”, where the italicized “*A*” represents P2 (Figure 5A), one may easily conclude that GlnR negatively regulates *ald* transcription *via* specific binding to P2 and acts as a roadblock to shut down the majority of *ald* transcription. In addition, binding of GlnR to *a2* site, which is downstream of P1 and P3 (Figure 5A), in theory blocks the transcription from P1 and P3 sites to a certain extent, which might well explain the reduction of *ald* transcription from P1 as well as P3 in response to nitrogen limitation.

## Discussion

So far, GlnR has been well characterized as a central regulator for governing global nitrogen metabolisms in several important actinomycetes, including *S. coelicolor*, *S. venezuelae*, *M. smegmatis*, *etc*. In most cases, GlnR exerts positive regulation on its target genes in response to nitrogen limitation; however, GlnR may sometimes act as a negative regulator (*e.g.* for *gdhA*) to coordinate the global metabolisms, helping the bacteria to cope with the environmental stress. *A. mediterranei* is an important actinomycete for rifamycin production and has been studied for decades in our lab. In *A. mediterranei*, GlnR has been characterized to positively regulate the transcription of *glnA* [11], *nas* operon [10] and *amtB* (unpublished data) under nitrogen-limited conditions; however, because the GDH activities do not change significantly in *A. mediterranei* (data not shown), transcription of *gdhA* is unlikely to be regulated. Interestingly, unlike most bacteria, *A. mediterranei* uses AlaDH pathway instead of the GDH pathway for ammonium fixation when the extracellular ammonium concentration is high. Therefore, a simple question raised is whether GlnR plays a repressor role in *ald* transcription in *A. mediterranei*, just as its homologues do in many other phylogenetically close actinomycetes? Based on the experiments exemplified in this study, we have proved that GlnR negatively regulates *ald* transcription and switches the ammonium assimilation route from the AlaDH pathway to GS/GOGAT pathway in response to nitrogen limitation, facilitating the bacterial adaption to the nutritional stress. Therefore, GlnR may be considered a global nitrogen regulator in *A. mediterranei*.

The GlnR binding *cis*-elements have been studied for years by several groups around the world. Through aligning several promoter sequences of the under-regulated genes, Fink *et al.* firstly proposed a 44-bp *S. coelicolor* GlnR binding motif [18]. Based on Fink's work, Tiffert *et al.* [6] later deduced a much shorter consensus sequence specific for GlnR binding, which is 22-bp, comprising of an “*a*-site” of gTnAc and a “*b*-site” of GaAAc with a fixed distance of 6 bases in between [6]. However, the GlnR binding cis-elements have been found to be extremely complicated and can be comprised of varied GlnR binding sites (from 0 to 6) in different target genes of different species [7], [14], [16], [19], [20]. *A. mediterranei* GlnR is not only structurally homologous to that of *S. coelicolor* GlnR [11] but also able to complement the glutamine auxotrophic phenotype of the *S. coelicolor glnR* null mutant *in trans* [21], and the two GlnRs are proposed to share much homogeneity in their binding DNA consensus sequences [10]. We ever characterized three essential GlnR binding sites (*a1*, *b1* and *b2*) in the *nas* promoter, which are of high similarity to the GlnR binding sites characterized in *S. coelicolor* [10]. Here, once again, we predict three GlnR binding sites in the promoter region of *ald* for further investigation. Notably, these sites in *ald* promoter are highly similar to those in *nas* promoter, in not only their primary DNA sequences but also their configurations. Although the mechanism for negative regulation of *ald* transcription by GlnR is obvious and clear, the in-depth depiction of the action of GlnR (*e.g.* how GlnR binds to these three sites) seems a mission impossible at present.

**Figure 6:**
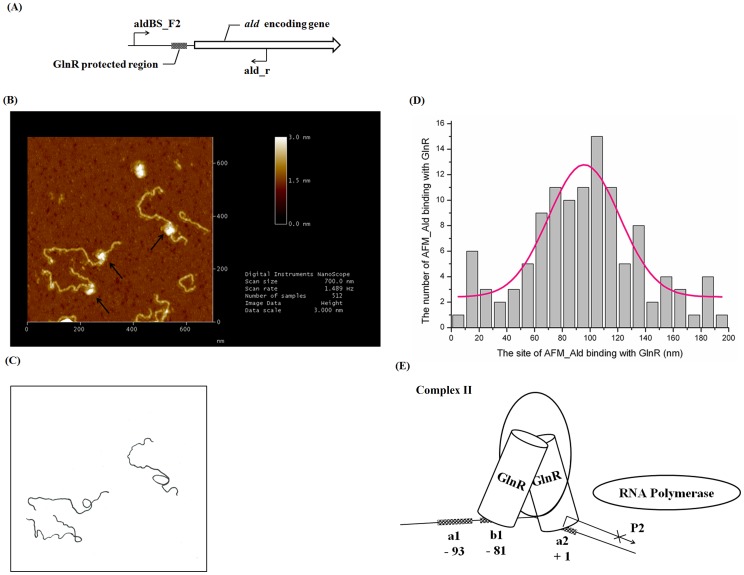
AFM analysis of the structural changes of *ald* promoter upon interaction with GlnR. (A) Schematic presentation of *ald* promoter used for AFM visualizing. (B) AFM images of fragment AFM_Ald with GlnR. Putative DNA loops were marked with black arrows. (C) Sketch of AFM_Ald fragments in Figure 6B, highlighting possible DNA loops. (D) Histogram of the distribution of GlnR binding sites on fragment AFM_Ald (n = 223 DNA molecules). The Gaussian centered 97 nm with a standard deviation of 42 nm. (E) Proposed mechanisms for GlnR-mediated negative regulation of *ald* transcription. GlnR finally binds *b1* and *a2* sites to form Complex II and blocks *ald* transcription initiated from *ald*P2, which contributes the majority of *ald* transcripts under nitrogen-excess conditions. A DNA loop may occur during the process. The numbers labeled show their relative distance to *ald*P2 site.

In the case of *nas* regulation, two complexes of CI and CII were found during the process of GlnR binding to *nas* promoter and two distinct steps were proposed. First, GlnR binds to *a1* and *b1* sites to form CI; then, on the basis of CI formation, GlnR further binds *b2* site to produce CII, which is required for the activation of *nas* transcription [10]. Although it has been known that *b1* and *b2* are required for the formation of CII, it is unclear whether GlnR binds to *a1* site in CII. Recently, based on the results of DSS-crosslinking assay and crystal structural analysis, Lin *et al.* demonstrated that GlnR mainly works as a homodimer [22], indicating only *b1* and *b2* sites are bound by a homodimer GlnR in the complex of CII. Due to the high similarities between the GlnR binding *cis*-elements in *nas* and *ald* promoters, we presume here a similar procedure for GlnR binding to the *ald* promoter, and GlnR may first bind to *a1*-*b1* sites and then to *b1–b2* sites with a final release of *a1* site (Figure 6E). As *b1* and *b2* sites are separated by 76 bps, the binding of a homodimer GlnR to *b1*–*b2* will therefore form a DNA looping (Figure 6E), which has been found widely involved in regulation of gene transcription, DNA recombination and replication [23]–[25].

To further test this hypothesis, we used Atomic Force Microscopy (AFM) to directly examine the change of DNA configuration after binding of GlnR to the *ald* promoter region (Figure 6A) *in vitro*. Probably due to the short distance between *b1* and *b2*, which in turn results in the formation of a small loop, and the aggregation of purified recombinant GlnR in solution, we failed to observe clear DNA loops after addition of GlnR (Figure 6B). However, dramatic change of the angles of the DNA molecules was detected (Figure 6B and 6C), indicating the existence of possible DNA loops. Besides, the position of bound GlnR on the DNA molecules was measured (Figure 6D) and a Gaussian fit was performed. The histogram was shown by a pink line, whose peak located at ∼100 nm (∼300 bps) from one end of the DNA molecules, in consistent very well with the predicted position of GlnR binding sites on the DNA molecules ( = 310 bps to one end).

Double-stranded DNA is semi-flexible and DNA fragments shorter than 150 bps are difficult to form a DNA looping [26]. As the space between *b1* and *a2* in *ald* promoter is only 76 bps, other protein(s) may probably participate in bending of the DNA between the sites and facilitate GlnR binding. Although possible loops were observed with merely GlnR addition in the *in vitro* AFM assay, the concentration of GlnR added was obviously higher than *in vivo* and thus may be unable to fully reflect the *in vivo* situation. And the suspects include a group of Nucleoid-Associated Proteins (NAPs), *e.g.* Fis (Factor for Inversion Stimulation), IHF (Integration Host Factor) and HU (Heat-Unstable Nucleoid Protein), which have been shown to be able to bend DNAs [27], [28] and are worth of further investigation.
